# National Implementation of an Electronic Patient-Reported Outcome Measures Program for Joint Replacement Surgery: Pilot Study

**DOI:** 10.2196/30245

**Published:** 2022-04-08

**Authors:** Emma L Heath, Ilana Ackerman, Michelle Lorimer, Sophia Rainbird, Grace O'Donohue, Andrew Brock, Stephen Graves, Ian Harris

**Affiliations:** 1 South Australian Health and Medical Research Institute Adelaide Australia; 2 School of Public Health and Preventive Medicine Monash University Melbourne Australia; 3 Australian Orthopaedic Association National Joint Replacement Registry Adelaide Australia; 4 South Western Sydney Clinical School Faculty of Medicine University of New South Wales Sydney Australia; 5 Whitlam Orthopaedic Research Centre Ingham Institute for Applied Medical Research University of New South Wales Sydney Australia

**Keywords:** cost, cost-benefit, online platform, patient-reported outcome measure, registry science, electronic data collection, electronic data, capture, joint replacement, PROMs, PROM, outcome measure, patient report, data capture, registry, surgery, operation, postoperative, surgical, data reporting, data collection

## Abstract

**Background:**

There is a global emphasis on expanding data collection for joint replacement procedures beyond implant attributes and progression to revision surgery. Patient-reported outcome measures (PROMs) are increasingly considered as an important measure of surgical outcomes from a patient’s perspective. However, a major limitation preventing wider use of PROMs data in national data collection has been the inability to systematically collect and share electronic information with relevant stakeholders in a comprehensive and financially sustainable manner.

**Objective:**

This study reports on the development of an electronic data capture and reporting system by a national registry for the collection of PROMs and the processes used to identify and overcome barriers to implementation and uptake. The study also aims to provide a cost breakdown of establishing and maintaining a nationwide electronic PROMs program.

**Methods:**

Between 2018 and 2020, 3 governance and advisory committees were established to develop and implement a PROMs pilot program nested within a nationwide joint replacement registry. The program involved electronic collection of preoperative and 6-month postoperative data for hip, knee, or shoulder replacement surgery from 44 Australian hospitals. Resource requirements for the program included a project manager, software developers, data manager, and statistician. An online platform was tested, refined, and implemented for electronic PROMs collection with scalability considered for future expansion to all Australian hospitals and additional data fields. Technical capabilities included different access for multiple user types, patient registration, automatic reminders via SMS text messages and email, online consent, and patient outcome real-time dashboards accessible for different user groups (surgeons, patients, hospitals, and project stakeholders).

**Results:**

During the PROMs pilot period there were 19,699 primary procedures undertaken with 10,204 registered procedures in the electronic system. This equated to 51.80% of people who had a joint replacement at participating hospitals during this period. Patient registration and data collection were efficient (20-30 seconds and 10-12 minutes, respectively). Engagement with the reporting dashboards (as a proportion of those who viewed their dashboard) varied by user group: 197/277 (71.1%) hospital administrators, 68/129 (52.7%) project stakeholders, 177/391 (45.3%) surgeons, and 1138/8840 patients (12.9%). Cost analysis determined an overall cost per patient of Aus $7-15 (approximately US $5-12) for 2 PROMs collections per joint replacement procedure once the program was established.

**Conclusions:**

Successful implementation of an orthopedic PROMs program with planned scalability for a broader national rollout requires significant funding and staffing resources. However, this expenditure can be considered worthwhile, given that collection and reporting of PROMs can drive health care improvement processes. Further consideration of strategies to improve stakeholder engagement with electronic reporting dashboards (particularly for patients and surgeons) will be critical to the ongoing success of a national PROMs program.

## Introduction

Orthopedic registries and health care service groups around the world are gradually expanding from routinely reported surgical data to include data on the self-reported health status of patients undergoing joint replacement surgery. Patient-reported outcome measures (PROMs) provide an indicator of surgical thresholds and treatment effects, with the potential to improve health care outcomes within the health care sector for these increasingly common and resource-intensive procedures. Guidelines have been produced regarding the types of PROMs that should be collected [1,2]. However, a major limitation preventing the wider use of PROMs data in population-based studies has been the inability to systematically collect and share electronic information with all relevant stakeholders in a comprehensive and financially sustainable manner.

There are major challenges in effectively collecting and utilizing patient-reported data in a population-based setting. Timing of data collection, optimal selection of PROMs instruments, consent and data collection processes, acceptable levels of data completeness, data security concerns, approaches to delivering stakeholder feedback, and the financial implications are some of the considerations. To date, orthopedic registries that collect PROMs have implemented varying approaches to address these considerations [1-3]. To ensure the effective implementation of a national PROMs collection program, there is a need to systematically design, develop, and test an approach that addresses all of these considerations and does so in a cost-effective manner [1].

In this paper, we report on the establishment of a bespoke electronic PROMs data collection and reporting system and the processes used to overcome identified barriers. This system was developed to facilitate a PROMs pilot program nested within the Australian Orthopaedic Association National Joint Replacement Registry (AOANJRR). We discuss project design, governance, resourcing, infrastructure, and multistakeholder engagement considerations. A comprehensive cost breakdown of establishing and maintaining an ongoing PROMs program nationally is provided to assist other researchers and clinicians who may be considering implementing a PROMs program in their jurisdictions.

## Methods

### Establishment of a PROMs Pilot Project

The primary purpose of the PROMs pilot program was to design, develop, and test a comprehensive approach to electronically collect PROMs data that could be effectively rolled out nationwide by the AOANJRR. Ongoing development of the purpose-built online platform, known as *RAPID* (Real-time Automated Platform for Integrated Data capture), continued throughout the pilot in response to learnings and stakeholder feedback. This continual refinement of processes and systems was undertaken to best position the PROMs program for a planned nationwide rollout.

### Governance, Funding, and Program Approval

Establishing project governance is considered a safeguard to protect patient data and to promote efficiency in the delivery of a PROMs program [4]. For this program, 3 separate groups were established to provide oversight. A Project Steering Committee was established and met quarterly to provide support and guidance for the program. The high-level support from the committee involved a multistakeholder approach including leadership across the Australian Orthopaedic Association (AOA), AOANJRR, orthopedic surgeons, partners, consumer representation, and project funders. An International PROMs Instrument subgroup was established to provide expert advice regarding selection of PROM instruments, additional items, and the timing of data collection [1]. Lastly, a PROMs Working Group was established and met regularly to provide expert advice around project implementation and troubleshooting support for practical issues identified during day-to-day operations.

### Ethical Approval

Relevant ethics and hospital governance approvals were obtained, consistent with local requirements [5]. The following Australian ethics committees approved the pilot program from which the data presented in this study were obtained: University of South Australia Human Research Ethics Committee (HREC; 200890), Sydney Local Health District Ethics Review Committee (Royal Prince Alfred Hospital Zone, HREC/18/RPAH/90), Calvary Health Care Adelaide HREC (18-CHREC-F004), Mater Misericordiae Ltd HREC (HREC/18/MHS/45), St Vincent’s Health and Aged Care HREC (HREC 18/14), University of Tasmania HREC (H0017292), Calvary Health Care Tasmania HREC (010418), St John of God HREC (1408), and Calvary Health Care Australian Capital Territory (25–2018). Furthermore, licensing requirements for use of the selected PROMs instruments were addressed.

### Staffing Requirements

Initially, a project manager oversaw the pilot program and a software developer commenced the design and build of the *RAPID* platform infrastructure. As data collection commenced, additional staffing resources were required and further team members were sourced (2 additional software developers, a data manager, and a statistician).

### Infrastructure: Software Development

During the initial design phase, available off-the-shelf software solutions were deemed not fit for the purpose of the Registry’s electronic PROMs collection. This was due to the lack of customizability, particularly pertaining to the layout of patient dashboards and reporting functionality, and concerns regarding ongoing costs and support. Developing the software in-house allowed for technical solutions to be developed for problems identified during the design, testing, and data collection phases. Lastly, *RAPID* was designed from the outset to be scalable for national data collection and included the capability to run multiple research projects simultaneously nested within the Registry.

**Figure 1:**
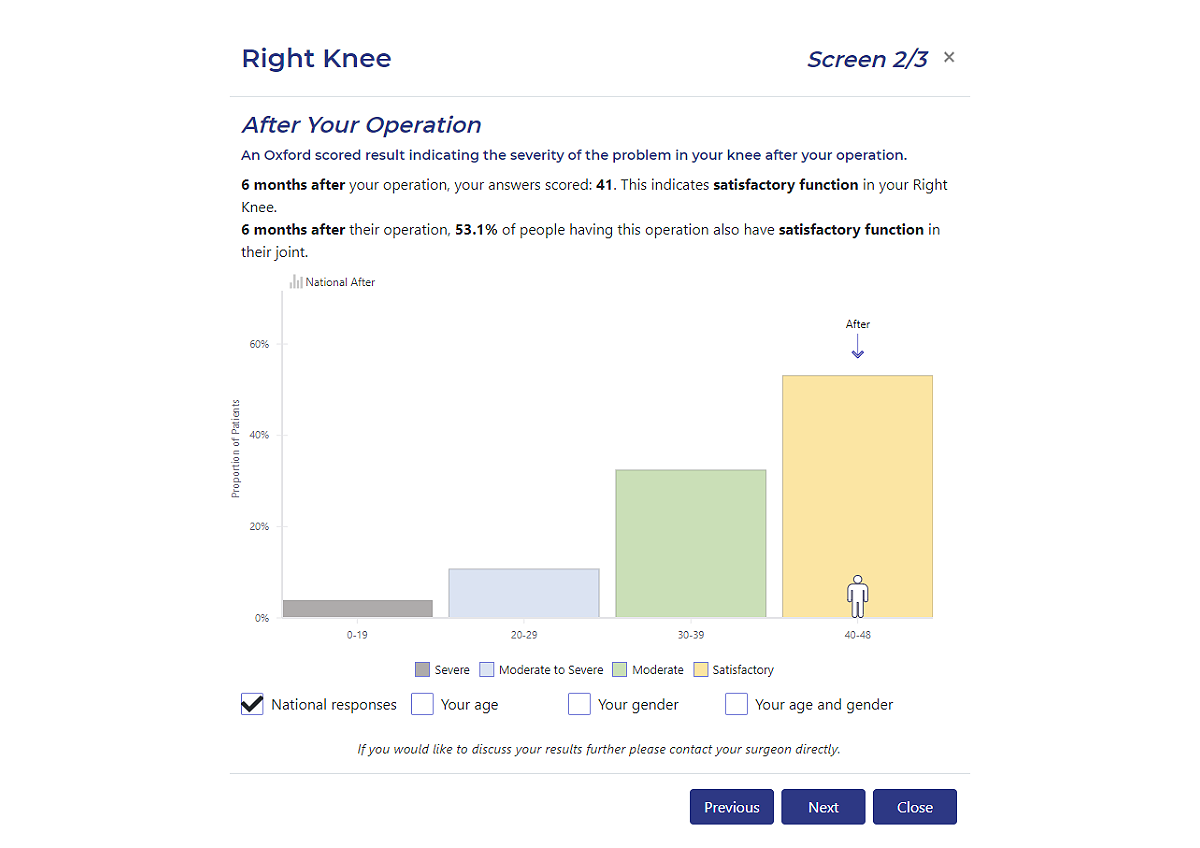
Example screenshot from a patient’s dashboard within the RAPID platform. RAPID: Real-time Automated Platform for Integrated Data capture.

*RAPID* included the ability for patients to provide online electronic consent, complete their preoperative/postoperative PROMs, as well as incorporate real-time dashboard reporting for patients to view their PROMs responses to compare their own responses as well as with national averages (Figure 1). Critical to the design of *RAPID* was to make the system usable for the specific patient population undergoing joint replacement who are predominantly elderly. Therefore, it was important to make the system as simple, user-friendly, and intuitive as possible. The number of “clicks” required was minimized, 1 question was displayed at a time, and, where possible, the PROMs questions were presented without the need for scrolling (this feature also enhanced viewing ease via a smartphone or other portable devices). Patients were provided with the option to go backward and review or change their answers if they wished to do so. However, the system did not allow for responses to questions to be left blank. Screenshot examples of *RAPID* can be found in 2 published AOANJRR reports [5,6].

**Figure 2:**
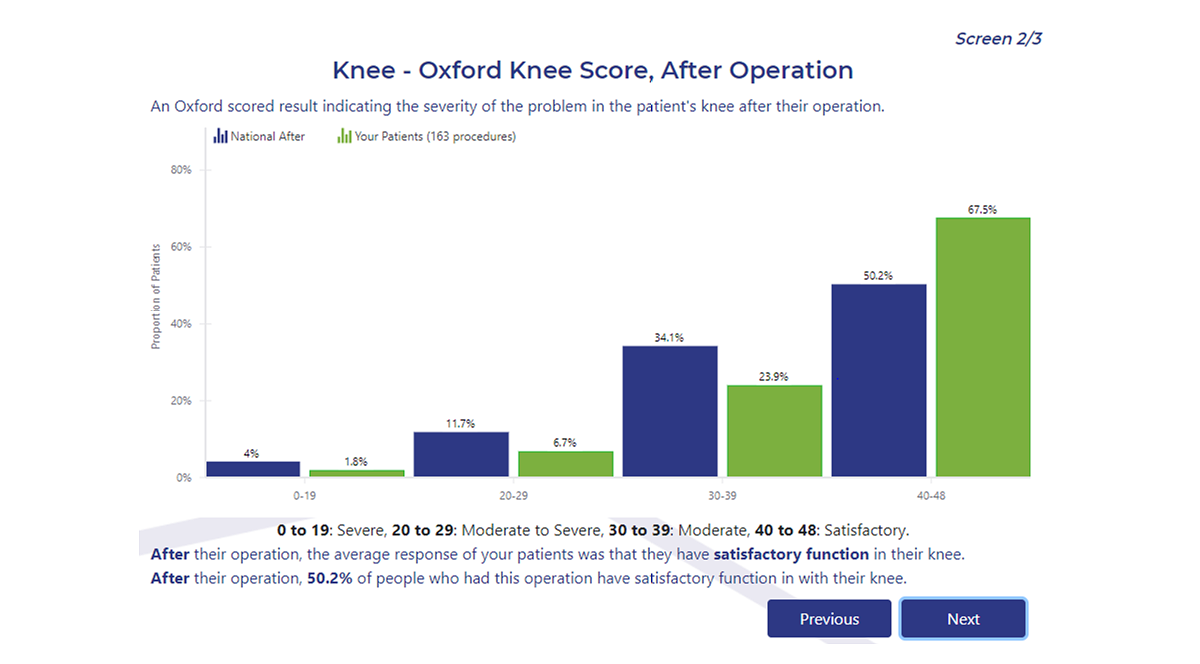
Example screenshot from a surgeon’s dashboard within the RAPID platform. RAPID: Real-time Automated Platform for Integrated Data capture.

Orthopedic surgeons had access to identified individual patient responses via *RAPID* when the patient consented to share their responses (12,236/12,874 [95.04%] preoperatively and 4653/4780 [97.34%] postoperatively). In addition, surgeons were able to download patient responses in a Microsoft Excel format. Dashboards were provided to all surgeons and these graphically displayed their patient recruitment data and PROMs outcomes, as well as comparisons with national averages (Figure 2). Surgeons were able to filter their patient cohort based on age or sex and compare different hospitals in which they operate. Designated hospital staff could also obtain similar data for the hospital cohort, following the provision of appropriate consent. Specific reporting dashboards were also designed for project stakeholders who were provided with aggregated national-level data.

An additional software feature of *RAPID* was the ability to integrate PROMs data collected by third parties so that some hospitals and surgeons could continue to collect data in their systems. This was deemed necessary due to the length of time some of the previous data collection systems were in place. The AOANJRR developed a standardized template for use by hospitals to send PROMs data via *RAPID*. Integration of the data into *RAPID* was conducted by the data management and statistical teams following a manual review of the data file. Any data discrepancies were queried and addressed prior to upload. However, this feature proved to be a highly resource-intensive process and opportunities to streamline this will be considered for the national rollout.

### Software Architecture and Security

The major components of the *RAPID* software architecture are a user interface built on the React web framework, with back end services using Spring Boot and a PostgreSQL database. These components were chosen due to their popularity within the software development community, breadth of documentation and community support, ease of development, and no licensing costs.

To securely log into *RAPID*, user access is role based for designated administrators, with access tailored depending on each role’s scope of responsibility within the system. For other users (such as surgeons and hospital staff), access is tailored based on their roles within various hospitals and other projects in *RAPID*. Additional security measures in *RAPID* include session timeouts, password expiries, minimum password strength limits, restrictions on password reuse, lockouts when the number of successive incorrect password attempts reaches a threshold, and a secure password hashing algorithm (PBKDF2). Access to the raw data (ie, in the database) is limited to data managers, statisticians, and information technology staff through firewall and access control measures.

## Results

### Engagement: Participation, Recruitment, Data Collection, and Quality

#### Hospital Participation

The pilot program included a broad cross section of public and private hospitals from all Australian states, of all sizes (small [<100 beds], mid-range [100-499 beds], and large [>500 beds]) and from urban and nonurban areas. Hospital representatives either volunteered to participate in the pilot PROMs program, were recruited following surgeon recommendation, or were invited to participate based on previous collaborative projects. In total, 44 hospitals provided pilot data.

#### Patient Recruitment and Hospital Training

Patients scheduled for primary or revision hip, knee, and shoulder replacement procedures were eligible. Patients under the age of 16 and patients with a cognitive impairment that impacted their ability to provide informed consent were excluded. Initial patient recruitment involved registration into *RAPID* via the collection of limited data including patient name, date of birth, postcode, contact details (1 or more of the following: email, mobile phone, and home phone), joint, side, surgeon (optional, if known), and hospital. Patients registered themselves or were registered by an administrator, via the *RAPID* platform. Administrators were provided training in *RAPID* by the project team and had on-site visits (or when not possible on-site, online training). Once registered, patients were able to provide electronic consent and then PROMs could be completed, either immediately or at a later time. Once registered, patients were provided with an electronic patient information sheet that detailed information about the Registry and the pilot program. A hard copy study information card was also provided to patients. If patients did not wish to participate, they were able to select “decline to participate” within *RAPID*, which deleted all identifying information recorded at registration from the system. However, only a small number of patients declined participation (944/14,890, 6.34%). SMS text messages and email reminders were effective in maintaining patient participation once patients were successfully registered in the electronic system [5]. Feedback provided by administrators and patients highlighted the efficiency of the patient registration and data collection system, indicating that it took 20-30 seconds to register a patient and 10-12 minutes for patients to complete their PROMs.

#### Data Collection

Automated email and SMS text message reminders were sent to patients via the *RAPID* platform as a reminder to complete their PROMs both preoperatively and 6 months postoperatively. The system allowed for a set number of reminders to be sent pre- and postoperatively, depending on the contact details provided at registration. Patients who had not completed their PROMs after 2 automated reminders appeared on a list for phone call follow-up. Phone call follow-up was trialed as part of the pilot and patients’ responses were entered directly into *RAPID* by a member of the phone call follow-up team. Comparisons of demographic information determined no substantial difference between patients who required phone call follow-up and those that responded to electronic reminders. Given this finding and associated costs, phone call follow-up was later phased out.

#### Data Quality

Throughout the data collection phase, the project manager communicated with orthopedic surgeons and personnel assisting with patient registration to ensure hospitals were well supported. A data summary was distributed regularly (containing both patient registration and procedure registration at each hospital), and hospitals were also directed to their *RAPID* reporting dashboards. If a hospital was identified as having low registration of patients, it was contacted so that processes could be reviewed and refined. Through this process, the AOANJRR determined that hospital registration improved over the pilot period, where more than 60.2% (441/733) of procedures undertaken at pilot hospitals were registered within the *RAPID* platform after 12 months of data collection, compared with 44.85% (2366/5275) in the first 3 months of data collection [5]. By examining learnings from hospitals that improved registration rates, the AOANJRR was able to implement the same processes to improve the performance of less satisfactory hospitals.

Surgical procedure forms received from each hospital were entered into the AOANJRR database as part of routine Registry processes. The patient information from the procedure form was then matched with the patient information in *RAPID*. This process assisted with determining the proportion of joint procedures registered at each hospital as well as identifying any data entry errors. It was also required for electronically triggering postoperative data collection reminders based on the procedure date. Reports were produced on patient registration and procedure registration rates at each hospital.

### Cost Analysis

#### Establishment Costs Associated With a PROMs Program

A multistakeholder approach to funding was established to foster a wide leadership base and to maximize engagement across the health care sector. Expenditure for the pilot PROMs program (n=14,890) was largely attributed to staffing costs as well as to the establishment of *RAPID*.

#### Staffing Costs

While the PROMs pilot was in the design and development phase, staffing costs were kept to a minimum by drawing on the expertise and experience of existing Registry staff. As the pilot developed and the scope increased, additional resources were required to establish PROMs collection within the pilot hospitals. This included the project manager (1.0 full-time equivalent [FTE]), software developers (2.5 FTE), data manager (0.8 FTE), and a statistician (0.6 FTE).

#### Information and Communications Technology Development Costs Associated With RAPID

A large portion of establishment costs was associated with the software development phase of *RAPID*. In addition to the information and communications technology (ICT) staffing costs, running costs included software licensing, servers (including nonproduction servers used for development and testing), and website certificates (Aus $27,000 [~US $20,000] over 2 years). Furthermore, to ensure secure storage of sensitive patient data, a security design review was conducted during the design phase and a follow-up software penetration test was conducted prior to launch (Aus $14,000 [~US $10,000] over 2 years). While these costs substantially contribute to the overall budget, the security of patient data is at the forefront of consideration in health care settings [7].

#### Trial of Tablets for Patient Registration Within Hospitals

A total of 70 tablet devices (specifically iPads) were purchased by the Registry and provided to 41 pilot hospitals to mitigate potential barriers relating to patients not having access to electronic devices and to encourage hospital participation. The total cost including providing cellular data for the devices was Aus $31,000 (~US $23,000). Data indicated that hospitals only intermittently provided patients with the tablets for patient self-registration and survey completion [5]. With this in mind, it has been decided that tablets will not be provided when the PROMs program rolls out nationally in Australia.

#### Cost of Telephone Follow-Up

During the pilot period, 1148/14,926 (7.7%) procedures registered in *RAPID* had a landline telephone only (predominantly patients aged ≥85 years) [5], precluding patient follow-up via SMS text messaging. Extrapolating the cost of Aus $65,000 (~US $48,000) for telephone follow-up in the pilot (involving about 15% [n=44] of available hospitals) to a broader national rollout produces an estimate exceeding Aus $450,000 (~US $336,000) annually. This was deemed to be financially unsustainable. Throughout the pilot and since its completion, AOANJRR has encouraged hospitals to obtain the contact details of a proxy individual (eg, a family member or friend) to receive reminders on the patient’s behalf and assist with the electronic completion of PROMs.

## Discussion

### Principal Findings

Owing to the success of establishing the PROMs pilot program within 44 Australian hospitals, a national rollout of the program is currently underway with ongoing government funding. A large portion of the funding attributed to the national rollout has been dedicated to staffing costs. The PROMs project manager, ICT software developer, data manager, and statistician have been retained. Two additional full-time project coordinators have subsequently been employed to facilitate expansion of the program from 44 hospitals to approximately 320 Australian hospitals, which requires significant engagement with each hospital site. The project coordinators are also responsible for continued engagement with the hospitals that participated in the pilot.

### Prediction of Anticipated Ongoing Cost of a PROMs Program Nationally

On completion of the national rollout program, it is anticipated that PROMs staffing costs may decrease with the potential to reduce to 1 project coordinator and 1 software developer while retaining the project manager, data manager, and statistician. Conversely, ICT costs are likely to gradually increase as the size of the *RAPID* platform expands to accommodate for increased accessing, processing, and storage of patient data. Increased server storage and security enhancements as the *RAPID* platform increases capacity are expected. Assuming PROMs data are collected on 40%-80% of joint replacement procedures (a range of ~44,000 and ~88,000 procedures; based on 110,000 primary hip, knee, and shoulder procedures for osteoarthritis and revision procedures for the 3 joints per annum), we estimate a cost per patient of Aus $7-15 (approximately US $5-12). We recognize that cost and governance processes differ between countries and jurisdictions, which makes direct comparisons for implementation in other settings challenging.

### Enablers and Barriers to Establishing a PROMs Program

#### Enabler: Successful Cost Minimization

Nesting the PROMs pilot project within the AOANJRR was key to successfully reducing costs as it allowed for shared staff resources throughout the lifecycle of the project, particularly in the planning and implementation phases of the project. This included utilizing the existing expertise, skills, and relationships of Registry staff to liaise with surgeons and clinicians directly at hospital sites. These relationships assisted with approval for hospitals to participate and reassuring surgeons regarding any process or security concerns. The well-established reputation of the AOANJRR allowed for confidence in data security by patients, surgeons, and stakeholders.

#### Enabler: Successful Online Electronic Data Collection

Electronic PROMs collection has proven to be a successful means of outcome data collection, as evidenced by other Registries, such as the Functional and Outcomes Research for Comparative Effectiveness in Total Joint Replacement (FORCE-TJR) Registry that has reported high levels of enrollment and data completion via electronic collection [8]. Our pilot has proven similar success in that of the patients who consented within *RAPID*, preoperative PROMs collection was obtained for 97.77% (12,871/13,164) of registered procedures, and 79.05% (4184/5293) of postoperative PROMs were completed [5]. Wilson et al [2] highlighted that paper-based questionnaires can be relatively easy for patients to complete, but issues persist with mail-out, having patients mail them back, following up on missing data, possible data entry errors, or duplications [2]. These issues were mitigated with the use of the *RAPID* platform. Another consideration is potential patient response differences when 1 question is viewed at a time (versus traditional paper-based collection) and the order in which the questions are answered. Some research has indicated that displaying 1 question at a time improves response rates; however, equivalence studies compared with paper-based collection would provide benefit to the orthopedic community [9]. In our pilot study, the small number of patients who were unable to access *RAPID* or could not be contacted via electronic means were encouraged to seek assistance from family or friends to complete their PROMs or to contact the Registry for assistance with online completion. In the pilot study, preoperatively, 10.34% (782/7562) of patients reported seeking assistance from a family member and 0.45% (34/7562) of patients reported seeking assistance from a friend. Technology is increasingly affordable and accessible with internet access in Australian private homes recorded at 86% [10]. Access and technological familiarity will likely continue to improve over time, including for older patients.

#### Enabler: Successful Customization of the RAPID Platform

The ability to adapt *RAPID* functionality following user feedback and respond quickly to address any identified issues was a critical enabler to improve engagement with hospital sites and optimize patient registration. Hospital staff feedback identified that patients in preadmission clinics are required to attend a variety of appointments during their preadmission review and this made it difficult to complete the survey on the supplied iPad in a single sitting. A system adjustment was then made to implement a “resume” function in *RAPID*. This allowed patients to exit *RAPID* part way through the survey and return to complete the survey within 14 days in their own time on any device.

Administrators identified during the pilot that the electronic consent format was proving onerous for patients as they had to click “I agree” to each consent statement (9 in total) before providing final consent. The system was updated so that the information sheet could be reviewed on a single page with patients consenting to participate in the study once the information was reviewed by pressing 1 button.

Registry staff identified that it would be helpful to generate additional PROMs completion reminders manually when patients requested. *RAPID* was updated to include a manual link that generated additional SMS text messages or email reminders on request.

Such enhancements proved important in facilitating data collection and reducing the burden on patients. This also underscores the importance of continuous software developer resourcing to support additional minor platform refinements, even after a project has launched.

#### Identified Barrier: Overburdening Hospital Staff and Patients

One of the main considerations for this program was to develop an electronic system that would not overburden hospital and administrative staff. Two pathways for patient registration were therefore developed; patient self-registration or registration of a patient by an administrative user. During the pilot, participant recruitment increased when the patient was registered by hospital staff. For the national implementation of this program, the Registry will continue to encourage the registration of patients by hospital staff.

Frequently reported barriers for patients to complete PROMs are the length of time to completion and difficulty using electronic devices [11]. Early reports from patients suggested that the time taken to complete the PROMs instruments was satisfactory (10-12 minutes for PROMs completion) [5]; however, further exploration of patient burden and preferences is necessary through seeking consumer representation [6].

#### Identified Barrier: Hospital Staff Turnover

Staff turnover at the hospitals was identified as an issue impacting patient recruitment. Registry staff were often unaware that a hospital staff member trained in using *RAPID* had left the hospital until a decrease in patient registration was identified. In these instances, it was identified that key project details were not communicated or understood when hospital staff handed over the task to new personnel. To alleviate this potential barrier, the Registry has implemented additional reporting strategies to monitor hospital recruitment as well as continuing to provide specific hospital training, education material, and induction documentation to meet the needs of new hospital staff.

### Conclusions

Successful implementation of a national PROMs pilot program with planned scalability for a national rollout can be achieved but requires significant funding and resources. However, the expenditure can be considered worthwhile given the high level of patient participation and the potential for PROMs data to drive improvement processes, improve care for patients, and optimize health care efficiency. Importantly, strategies to improve stakeholder engagement with electronic reporting dashboards (particularly for patients and surgeons) need to be investigated further, as this will be critical for the ongoing success of a national PROMs program.

## Abbreviations

AOA: Australian Orthopaedic Association
AOANJRR: Australian Orthopaedic Association National Joint Replacement Registry
FORCE-TJR: Functional and Outcomes Research for Comparative Effectiveness in Total Joint Replacement
FTE: full-time equivalent
HREC: Human Research Ethics Committee
ICT: information and communications technology
PROM: patient-reported outcome measures
RAPID: Real-time Automated Platform for Integrated Data capture
